# Prognostic and Predictive Biomarkers of Oligometastatic NSCLC: New Insights and Clinical Applications

**DOI:** 10.1016/j.jtocrr.2024.100740

**Published:** 2024-10-17

**Authors:** Mandy Jongbloed, Martina Bortolot, Leonard Wee, Jarno W.J. Huijs, Murillo Bellezo, Rianne D.W. Vaes, Frank Aboubakar Nana, Koen J. Hartemink, Dirk K.M. De Ruysscher, Lizza E.L. Hendriks

**Affiliations:** a Department of Pulmonary Diseases, GROW Research Institute for Oncology and Reproduction, Maastricht University Medical Center+, Maastricht, The Netherlands; b Department of Medicine (DMED), University of Udine, Udine, Italy; c Department of Radiation Oncology (Maastro Clinic), GROW Research Institute for Oncology and Reproduction, Maastricht University Medical Center+, Maastricht, The Netherlands; d Department of Pneumology, Cliniques Universitaires Saint Luc, Brussels, Belgium; e Department of Surgery, Netherlands Cancer Institute, Antoni van Leeuwenhoek Hospital, Amsterdam, The Netherlands; f Department of Thoracic Oncology, Leiden University Medical Center, Leiden, the Netherlands

**Keywords:** NSCLC, Oligometastatic disease, Biomarkers, Prognostic, Predictive

## Abstract

This review discusses the current data on predictive and prognostic biomarkers in oligometastatic NSCLC and discusses whether biomarkers identified in other stages and widespread metastatic disease can be extrapolated to the oligometastatic disease (OMD) setting. Research is underway to explore the prognostic and predictive value of biological attributes of tumor tissue, circulating cells, the tumor microenvironment, and imaging findings as biomarkers of oligometastatic NSCLC. Biomarkers that help define true OMD and predict outcomes are needed for patient selection for oligometastatic treatment, and to avoid futile treatments in patients that will not benefit from locoregional treatment. Nevertheless, these biomarkers are still in the early stages of development and lack prospective validation in clinical trials. Furthermore, the absence of a clear definition of OMD contributes to a heterogeneous study population in which different types of OMD are mixed and treatment strategies are different. Multiple tissue-based, circulating, and imaging features are promising regarding their prognostic and predictive role in NSCLC, but data is still limited and might be biased owing to the inclusion of heterogeneous patient populations. Larger homogeneous and prospective series are needed to assess the prognostic and predictive role of these biomarkers. As obtaining tissue can be difficult and is invasive, the most promising tools for further evaluation are liquid biopsies and imaging-based biomarkers as these can also be used for longitudinal follow-up.

## Introduction

NSCLC is the leading cause of cancer-related mortality, primarily attributed to the advanced stage at which patients manifest symptoms.^1^ Approximately 50% to 60% of patients have metastatic disease at diagnosis and historically these were all deemed ineligible for curative intent therapy.^2^ A specific subgroup within the metastatic spectrum consists of oligometastatic NSCLC. The term "oligometastases" described by Hellman and Weichselbaum in 1995 characterizes a state of limited metastatic disease (in recent clinical trials defined as up to three to five metastases in up to three organs) wherein the use of local radical therapy (LRT) may result in long-term disease control or even cure.^3^^,^^4^ Over the past decades, advancements in diagnostic imaging, surgical and radiation techniques (minimally invasive surgery and stereotactic body radiotherapy [SBRT]), combined with the promising survival outcomes shown by various (often retrospective) studies, resulted in an increased interest in treating oligometastatic disease (OMD).^5^, ^6^, ^7^, ^8^, ^9^, ^10^ Although clinical guidelines recommend the addition of LRT to synchronous oligometastatic NSCLC responding to systemic therapy, this is based on limited evidence.^11^, ^12^, ^13^ Furthermore, as immune checkpoint inhibitors (ICIs) or tyrosine kinase inhibitors (TKIs) based strategies have become the preferred first-line treatment according to the tumor’s molecular profile, their use may offer advantages to patients with OMD undergoing LRT.^14^^,^^15^

Importantly, the absence of a clear definition of OMD contributes to variations in patient selection for clinical trials or OMD treatment in clinical practice, resulting in different treatment courses and survival. This complexity is further increased by challenges in distinguishing different forms of oligometastases, such as metachronous and synchronous OMD. Guckenberger et al.^16^ proposed a structured multidisciplinary consensus, incorporating the expertise of both the European Society for Radiotherapy and Oncology and the European Organisation for Research and Treatment of Cancer, to classify nine subgroups of OMD, aiming for a nuanced characterization of each scenario. Nonetheless, the existing definitions of OMD are exclusively based on the number of metastases, neglecting the influence of for example the aggressiveness, volume, and genomic landscape of the tumor. Biomarkers that aid in defining true OMD and predicting survival and safety outcomes are needed for patient selection for oligometastatic treatment, and to avoid futile treatments in the patients that will not benefit from LRT. As reported in the interim analysis of the NRG-LU002 phase II/III trial (NCT03137771), adding LRT to maintenance systemic therapy in patients with oligometastatic NSCLC (≤3 extracranial metastases after induction systemic therapy, the majority receiving ICI, N = 215) did not significantly improve the two-years progression-free survival (PFS) rate (52% in the maintenance systemic therapy group versus 40% in the LRT and maintenance systemic therapy combination group, *p* = 0.66). This suggests that LRT in a biomarker unselected group does not improve PFS and even causes additional toxicity, which underlines the need for biomarkers to optimize patient selection.^17^ Research is ongoing to explore the biological attributes of tumor tissue, circulating cells, the tumor microenvironment (TME), and imaging findings as potential predictive and prognostic biomarkers for oligometastatic NSCLC.^18^ Nevertheless, these biomarkers are still in the early stages of development, lacking prospective validation in clinical trials.

We reviewed the existing literature on biomarkers in oligometastatic NSCLC and explored the future directions for this emerging field.

## Tumor-Sample-Based Biomarkers

### Genomic Alterations

In personalized medicine, genomic analysis of the tumor is the cornerstone to guide the treatment strategy. Genomic analyses are evolving rapidly and an increasing number of actionable genomic alterations are being discovered.^18^ A drawback of genomic analysis of a tissue biopsy is the fixed aspect of the assessment of the genomic stature, missing the potential heterogeneity in the primary tumor versus the metastasis. In addition, systemic therapy by itself can cause heterogeneous resistance mechanisms which could be associated with further treatment unresponsiveness and survival.^19^, ^20^, ^21^

Several genomic alterations are negative prognostic factors for survival in metastatic NSCLC such as *KRAS,* Small Mothers Against Decapentaplegic and fibroblast growth factor receptor 3 mutations, and neurogenic locus notch homolog protein signaling. These alterations have not been evaluated in oligometastatic NSCLC, but for some other alterations, limited prognostic data is available. In a retrospective series (N = 9), transforming growth factor beta receptor 2 and Rho-associated coiled-coil kinase isoforms 2 mutations were considered prognostic for OMD as they were associated with a favorable clinical course.^22^ A retrospective study (N = 98) evaluated the prognostic impact of genetic alterations in patients with synchronous OMD (sOMD) NSCLC with 3 or fewer metastatic lesions (n = 77) and compared these to polymetastatic NSCLC (n = 21).^18^ Alterations in anaplastic lymphoma kinase*,* v-erb-b2 avian erythroblastic leukemia viral oncogene homolog 2*,* mixed-lineage leukemia 4*,* phosphatidylinositol 3-kinase complex, and topoisomerase II alpha were less frequently detected in oligometastatic compared to polymetastatic NSCLC, suggesting that stage IV tumors harboring those mutations are more likely to have polymetastatic behavior.

In early and advanced stage NSCLC, genomic alterations are presumed to be predictive biomarkers for therapy with TKI, and in these cases, a TKI is recommended.^11^^,^^22^ Exploring the predictive impact of these alterations could be interesting in OMD NSCLC^22^^,^^23^ and help in selecting the best systemic upfront treatment. In a subgroup analysis of a phase II trial (N = 49), the presence of an *EGFR* mutation or anaplastic lymphoma kinase rearrangement was predictive of a reduced risk of death in patients with sOMD NSCLC treated with first-line TKI (n = 8), but no subgroup analyses with the effect of the addition of LRT were performed because of the small population.^5^ In the phase III SINDAS trial (NCT02893332, N = 133), patients with *EGFR*-mutated synchronous OMD NSCLC treated with first-line TKI therapy were randomized to upfront LRT (using radiation therapy [RT]) versus no LRT.^24^ Although *EGFR* mutation type (exon 19 versus exon 21 mutation) was an independent predictor of overall survival (OS) (*p* = 0.001) it was regardless of receiving upfront LRT. Therefore, *EGFR* mutation type can not be considered as a predictive biomarker for the effect of LRT in the specific OMD setting as an *EGFR* exon 19 mutation is also associated with better survival in patients treated with TKI in advanced *EGFR*-mutated NSCLC.^25^

### Tumor Mutational Burden

High tumor mutational burden (TMB) (≥10 mutations/megabase) seems to be a predictive biomarker associated with improved survival outcomes in patients with NSCLC and other solid tumors treated with ICI.^14^^,^^26^ Conversely, evidence of its prognostic role is lacking, although a meta-analysis including ten retrospective and prospective studies (N = 2520) reported that the prognostic impact of TMB was limited in early-stage NSCLC.^27^ Few data are available about the predictive and prognostic role of TMB in oligometastatic NSCLC or other tumor types. In metastatic NSCLC, a retrospective study (N = 143) evaluated the differences in baseline characteristics between oligo-acquired resistance (≤3 lesions of disease progression) and systemic acquired resistance in patients treated with first-line PD-(L)1 inhibition. Baseline TMB was higher in patients with oligo-acquired resistance compared to systemic acquired resistance (9.8 mutations/megabase versus 5.3 mutations/megabase, *p* = 0.007) occurring on treatment with first-line PD-(L)1 inhibition, suggesting that baseline TMB could be associated with a pattern of oligoprogression.^28^ Nevertheless, data in this setting is limited, and future studies including a larger homogenous cohort of oligometastatic NSCLC are needed. Research should focus not only on the detection of TMB in OMD but also on the correlation between TMB and prognosis and on the predictive role of TMB for long-term disease control during ICI treatment.

### MicroRNA

*MicroRNAs* (*miRNAs*) play a role in developing metastasis by promoting the invasiveness and motility of the tumor.^29^ After Lussier et al.^30^ identified that *miRNA-200c* was responsible for the transition from an oligo- to polymetastatic phenotype, *miRNAs* were further evaluated in OMD.

In NSCLC, *miRNA* expression patterns manifest differently in patients with metastatic and non-metastatic disease and this could help in distinguishing patients with true OMD from those that are prone to develop widespread metastases.^31^^,^^32^ Specific *miRNAs* are proposed to function as a prognostic biomarker, but they could also be predictive for treatment response. This has been shown for widespread metastatic NSCLC treated with platinum-based chemotherapy in which downregulation of *miR-1273a* was associated with poor survival.^33^^,^^34^ A retrospective study analyzed *miRNA* expression patterns from resected lung metastasis samples from patients with oligometastatic NSCLC and five or fewer synchronous metastases treated with curative intent.^29^ *MiRNA* patterns associated with tumor-suppressive activity were downregulated in the tumors of patients who experienced rapid disease progression and poor survival. Another study screened 63 lung metastases (NSCLC, soft tissue sarcoma, osteosarcoma, melanoma, transitional cell carcinoma, and uterine sarcoma) samples after resection of patients with either OMD (n = 39) and polymetastatic disease (n = 24) and found that *miR-329*, *miR-431*, *miR-485-3p*, *miR-654-5p*, *miR-655*, *miR-887* and *miR-891a* were associated with OMD.^35^

Although an exploratory analysis of a study showed the possible prognostic and predictive role of 3 *miRNAs* (*miR-23b*, *miR-449a*, and *miR-449b*) in patients with OMD (n = 17) regardless of histology, no robust evidence is available for their use as biomarkers.^36^

A limitation is that *miRNA* expression can be affected by multiple factors, for instance, endogenous compounds (cytokines and hormones), immune system activation, physical activity, diet, and receiving systemic therapy. Moreover, there is no standardized strategy for sample and data collection, which could lead to conflicting results.^33^^,^^37^

### Programmed Death-Ligand 1 Expression

The level of expression of programmed death-ligand 1 (PD-L1), displayed as the tumor proportion score, is used for the decision to start with first-line ICI monotherapy or ICI-chemotherapy in advanced NSCLC.^11^^,^^12^ PD-L1 expression in early and advanced stage NSCLC seems to be prognostic for poor survival in patients treated without an ICI-based strategy.^38^, ^39^, ^40^ Conversely, across all disease stages, an increased PD-L1 expression is generally associated with improved survival outcomes after treatment with ICI; PD-L1 is currently the only predictive biomarker used in clinical guidelines.^41^^,^^42^

The prognostic or predictive value of PD-L1 expression in oligometastatic NSCLC is unknown, and it is unclear whether the data from the polymetastatic or non-metastatic setting can be extrapolated to OMD. A small retrospective study (N = 67, ≤3 metastases) focused on the prognostic and predictive value of PD-L1 expression in patients with oligometastatic NSCLC. With all the caveats of a retrospective study, PD-L1 expression (≥25%) was associated with poorer survival in the cohort of patients not treated with PD-L1 inhibitors, whereas survival improved in those treated with ICI.^43^ Nevertheless, the poor survival could also be because those patients were not treated with an ICI-based strategy, and larger prognostic trials are needed to assess the prognostic and predictive effects of PD-L1 expression in patients with OMD NSCLC.

### Tumor-Infiltrating Lymphocytes

Cluster of differentiation (CD) 8+, CD4+, and CD3 tumor-infiltrating T-cells (TILs) are linked to better survival in NSCLC and CD8+ T-cells seem to have a positive prognostic value. Tumors containing infiltrated CD8+ T-cells are proposed to have high levels of PD-1 and T-cell immunoglobulin- and mucin-domain-containing molecule-3 and are more sensitive to ICI.^44^ Higher expression of inhibitory receptors by CD4+ and CD8+ T cells and the presence of immunosuppressive cells in the TME could be associated with resistance to ICI.^45^ As with PD-L1 and TMB, TILs have a difference in spatial distribution in the TME and this could influence survival outcomes.^46^ In clinical practice, there are no standard methods or cutoff values available to assess the effect of TILs.^47^

Currently, there is no data on the prognostic value of TILs in oligometastatic NSCLC. A small retrospective single-center study (N = 29) investigated the baseline (before any treatment) and surgical samples of patients with locally advanced (n = 23) or oligometastatic (n = 6) NSCLC who had received neoadjuvant chemo-ICI followed by surgery.^48^ A sub-analysis of available baseline and surgical samples (n = 10), suggested that neoadjuvant chemoimmunotherapy results in elevated CD3+ and CD8+ TILs in the stroma and tumor after neoadjuvant treatment, implying that neoadjuvant chemoimmunotherapy causes immune activation. Furthermore, high infiltration of CD8+ count in the stroma was associated with a beneficial pathological response, indicating the possibility of TILs as a predictive factor. In addition, CD8+ TILs aggregated in the tumor are more likely to have a modest pathological response compared with the CD8+ TILs in the stroma. Notwithstanding, the study population was small and there were no subgroup analyses performed for OMD.

## Circulating Biomarkers

Liquid biopsy is an emerging noninvasive method of analyzing peripheral blood samples to capture the genomic landscape and overcome the spatial and temporal heterogeneity that tissue biopsy is lacking. Liquid biopsies may identify patients with residual subclinical disease after systemic therapy and after induction systemic therapy followed by LRT.^49^, ^50^, ^51^ Numerous cancer-related molecules such as cell-free DNA, circulating tumor DNA (ctDNA), blood TMB, allelic variants from ctDNA, circulating tumor cells, neutrophil to lymphocyte ratio, and soluble PD-L1 can be characterized in liquid biopsies.^50^

### ctDNA

Using ctDNA, minimal residual disease after LRT can be quantitatively detected in solid tumors, which could be of importance as a predictive biomarker.^50^ In NSCLC, the negative prognostic value of detecting minimal residual disease using ctDNA has been extensively reviewed.^52^^,^^53^ With the current sensitivity of DNA detection, advanced stages are more likely to have detectable ctDNA in the bloodstream.^54^^,^^55^ Moreover, ctDNA can also be used to evaluate treatment efficacy as patients who clear ctDNA after curative or palliative treatment have better survival outcomes compared with those who do not.^56^, ^57^, ^58^ A limitation of ctDNA detection is that multiple methodological approaches can be used and differ in sensitivity and specificity, which could lead to inaccurate interpretation of results.^54^^,^^55^ Moreover, biological features of ctDNA are mostly unexplained and it is unknown which factors influence ctDNA shedding.^54^

In a large multicenter retrospective study (N = 309), ctDNA was assessed in the subgroup (n = 230) of patients with oligometastatic NSCLC treated with RT. Pre-RT ctDNA was found to be prognostic and was detected in 74%.^49^ Detectable compared to undetectable pre-RT ctDNA was associated with worse survival outcomes, with a median PFS of 5.4 versus 8.8 months, respectively (hazard ratio [HR]  =  1.57, 95% confidence interval [CI]: 1.15–2.13, *p* = 0.004); median OS was 16.8 versus 25.0 months (HR =  1.65, 95% CI: 1.05–2.61, *p* = 0.030). High ctDNA levels, assessed by either variant allele frequency or the mutational burden, were associated with a higher risk of disease progression and poor survival.^49^

In a phase II prospective trial (n = 31) including patients with oligometastatic NSCLC (≤3 metastases), changes in ctDNA induced by LRT were investigated.^51^ Patients were randomized in the LRT arm or maintenance therapy/observation arm. Patients in the LRT arm had decreased ctDNA at early follow-up compared with the maintenance therapy/observation arm. Six patients with serial ctDNA analysis had an increase in ctDNA detection which preceded clinical progression on imaging by 6.7 months. Larger prospective trials are needed to determine ctDNA as a prognostic and predictive factor in patients with OMD.

An upcoming placebo-controlled randomized phase II study (ICARS, NCT06219317) will examine whether maintenance cemiplimab after induction cemiplimab (with or without chemotherapy) and LRT improves PFS in patients with sOMD NSCLC. Circulating tumor DNA analysis will be performed at baseline, after induction, after LRT, at consolidation, at the end of treatment, and at progressive disease, which evaluates the strength of ctDNA analysis as a prognostic and predictive factor.

### Other Circulating Biomarkers

As cytokines and chemokines play a part in antitumor immune responses, they could be of interest as prognostic and predictive biomarkers. Changes in peripheral cytokines induced by LRT were assessed in a phase II prospective trial (n = 31) including patients with oligometastatic NSCLC (≤3 metastases).^51^ Elevated interleukin-1α concentration at baseline was associated with longer PFS and OS. Interleukin-1α is secreted by macrophages and starts the inflammation process, leading to antitumor activity. Furthermore, this study suggests that RT decreases the diversity of peripheral T cell receptors, followed by clonal expansion of these specific T cell receptors.

These findings are consistent with the results of a phase II trial of men with recurrent low-volume (1–3 metastases) metastatic hormone-sensitive prostate cancer treated with stereotactic body ablative radiotherapy (SABR) (N = 49).^59^ A T cell receptor clonal expansion was noticed 90 days after LRT, but not in the observational group (*p* < 0.024). A high percentage of regulatory T-cells was assessed in the blood of a small group of patients (n = 4) who underwent lung wedge resections for colorectal cancer oligometastases (1–3 nodules) when compared to healthy donors; the patients also displayed a lower percentage of CD16-56 positive cells.^60^

## Image-Based Biomarkers

### Radiomics and Artificial Intelligence

In the emerging era of radiomics, advances in artificial intelligence are being used to quantify tumor characteristics and assess their possible prognostic or predictive role.^61^^,^^62^ Radiomics holds promise for guiding therapy as it offers a view of the entire tumor in vivo, and potentially provides quantitative measurements of disease development, progression, and also for response to treatment. Clinical imaging is often noninvasive and frequently repeated during treatment in routine practice. Treatment responses are assessed on imaging by measurement of the sum of tumor, lymph nodes, and metastasis diameters before and after therapy on the basis of the Response Evaluation Criteria in Solid Tumors version 1.1.^63^ Defining phenotypic aspects of tumors beyond Response Evaluation Criteria in Solid Tumors (e.g., metrics of shape complexity, textures related to tumor heterogeneities, markers related to necrosis or cell infiltration, and so on) by using imaging-based radiomic features could be a potential non-invasive prognostic and predictive biomarker, as recent studies have shown^61^^,^^64^, ^65^, ^66^, ^67^ in NSCLC in the non-OMD setting. Voxel-by-voxel–based radiomics could help identify tumor heterogeneity by determining tumor subregions with comparable radiologic phenotypes (habitats) and may be later correlated with genetic variations.^68^

In a small retrospective study (N = 79) with oligometastatic NSCLC (≤3 metastases), handcrafted radiomic signatures on the basis of pre-treatment scans were added to a model with standard clinical prognostic factors, strengthening the prediction performance of the model for OS.^69^ In an ongoing phase II randomized controlled trial (ImmunoSABR), the efficacy and safety of SABR combined with immunotherapy (L19-IL2) is compared with standard-of-care treatment in patients with limited metastatic NSCLC. Using radiomics, the prognostic value of phenotypic differences will be assessed and the predictive power of tumor hypoxia for treatment response will be evaluated.^70^ Another ongoing phase III randomized controlled trial will compare the radiomics of pre-treatment and post-treatment computed tomography (CT) scans of patients with oligometastatic NSCLC treated with standard maintenance therapy with or without local consolidative RT.^71^

In the future, studies should focus on the use of radiomics for the detection of prognostic factors or to strengthen prediction models for survival in patients with oligometastatic NSCLC.

### Tumor Burden

Fluorodeoxyglucose positron emission tomography (FDG-PET) scans can be used for quantification of the metabolic disease burden by measuring the metabolic tumor volume (MTV), total lesion glycolysis (TLG), and standardized uptake value (SUV).^62^ In early and advanced stages of NSCLC, MTV, and TLG are proposed to be prognostic and predictive factors for survival and treatment outcomes.^72^, ^73^, ^74^, ^75^, ^76^, ^77^, ^78^ The prognostic value of maximum SUV is questionable as a single-pixel value might not reflect the tumor biology.^79^

A small retrospective study (N = 67) of sOMD NSCLC (n = 50) reviewed the prognostic value of metabolic disease burden on pre-treatment FDG-PET-CT scans in patients who had chemotherapy or surgery upfront before receiving SABR at all disease sites.^80^ Both MTV (HR = 2.42, *p* = 0.009) and TLG (HR = 2.14, *p* = 0.004) were predictive factors for shorter OS, and maximum SUV and number of lesions were not. Nevertheless, this study was performed before the introduction of ICI, and larger prospective studies are needed. Another retrospective study (N = 157) included patients with NSCLC and synchronous solitary bone metastasis and analyzed the prognostic power of the MTV of the thorax, bone, and whole body.^81^ The results suggest that whole-body MTV (HR = 1.003, 95% CI: 1.00–1.01, *p* = 0.018) and thorax MTV (HR = 1.003, 95% CI: 1.00–1.01, *p* = 0.023) are independent prognostic factors. Nevertheless, the patient population was selective as only patients with a solitary bone metastasis were included and the chosen treatment strategy was unknown.

It is hypothesized that analyzing metabolic tumor burden on pre-treatment FDG-PET-CT scans could predict the radioresistance of oligometastases treated with SBRT, which could help in selecting aggressive LRT or systemic treatment.^82^ This is on the basis of the assumption that the Warburg effect (aerobic glycolysis with the formation of lactate) increases radioresistance and promotes metastasizing. Nevertheless, only five studies examining the predictive power of pretreatment FDG-PET-CT scan metrics in OMD treated with SBRT are available and their preliminary data suggest that FDG-PET scans could be of use in predicting outcomes after SBRT of oligometastases or characterize radiosensitivity.^82^

Tumor burden can also be quantified by measuring the total tumor volume on a CT scan. Large tumor volume seems to be associated with poor survival and treatment outcomes in early and advanced stages of NSCLC, nevertheless, study designs are heterogeneous.^83^, ^84^, ^85^, ^86^, ^87^, ^88^ In a small retrospective study (n = 43) of patients with sOMD NSCLC (≤3 metastases) treated with concurrent chemoradiation, high total tumor volume (planning target volume ≥ 639cc) and absence of surgery of the primary tumor were predictors of poor OS. Nonetheless, limitations were the retrospective nature and the small and selected population as most of the patients had solitary brain metastasis.^9^ Another retrospective study measured tumor size in patients with NSCLC treated with SBRT for synchronous oligometastases (n = 66), oligoprogressive disease (n = 20), or local control of dominant tumors (n = 22). The study suggests that a tumor size larger than 4 cm (HR = 3.24, 95% CI: 1.58–6.65, *p*  =  0.001) was predictive of a higher rate of local failure. Nonetheless, this study was conducted before the introduction of ICI, and the population was heterogeneous as patients received previous systemic therapy.^89^ In the future, more studies should investigate the prognostic power of total tumor volume.

## Discussion

In recent years, OMD has become an emerging area of interest owing to the possibility of achieving longer survival outcomes for patients who were historically considered uncurable. Clinical guidelines recommend adding LRT to systemic therapy for patients with oligometastatic NSCLC, but considerable variations in daily clinical practice are still present, resulting in different treatment courses and survival.^11^, ^12^, ^13^

Biomarkers that help define true OMD and predict survival and safety outcomes are needed for patient selection for oligometastatic treatment, and to avoid futile treatments in the patients that will not benefit from LRT. As summarized above, mainly small retrospective studies or exploratory analyses have been conducted, from which it is difficult to define the strength of the potential biomarker, and whether the biomarker is either predictive, prognostic, or both. Furthermore, the lack of a clear definition of OMD contributes to a heterogeneous population in which different types of OMD (synchronous and metachronous) are mixed and treatment strategies are different (LRT with or without systemic therapy). As a result, it is difficult to distinguish between the subtypes of OMD and make a solid conclusion for that specific setting. The Research and Treatment of Cancer consensus definitions could help unify this and prevent selection bias and assure adequate baseline staging.^4^^,^^16^

**Figure 1:**
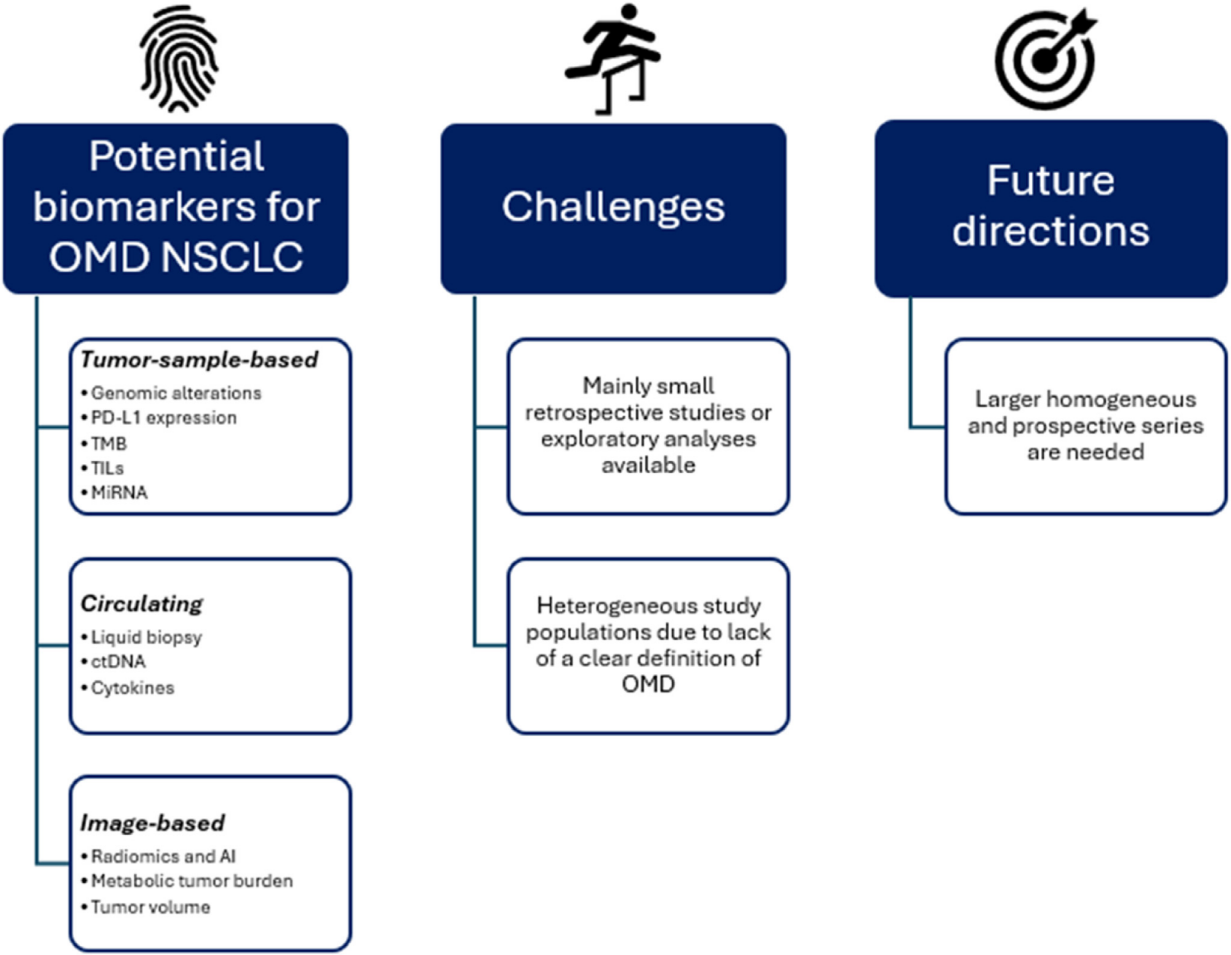
Potential biomarkers for oligometastatic NSCLC, current challenges in identifying biomarkers, and future directions. OMD, oligometastatic disease; PD-L1, programmed death ligand-1; TMB, tumor mutational burden; TILs, tumor-infiltrating lymphocytes; *miRNA*, *micro RNA*; ctDNA, circulating tumor DNA; AI, artificial intelligence.

In the future, larger homogeneous series with a clear definition of OMD are needed to determine the prognostic but also the predictive effect of the potential biomarkers in oligometastatic NSCLC (Fig. 1). As obtaining tissue can be difficult and is invasive, the most promising tools for further evaluation are liquid biopsies and imaging-based biomarkers as these can also be used for longitudinal follow-up.

## Conclusion

Multiple tissue-based, circulating, and imaging biomarkers hold promise in their prospective and predictive role in NSCLC, nevertheless, data on OMD NSCLC is scarce and on the basis of heterogenous populations without a clear definition of OMD. Larger homogeneous and prospective series are needed to assess the prognostic and predictive role of these biomarkers in oligometastatic NSCLC.

## CRediT Authorship Contribution Statement

**Mandy Jongbloed:** Conceptualization, Writing - original draft, Writing - review and editing.

**Martina Bortolot:** Writing - original draft, Writing - review and editing.

**Leonard Wee:** Writing - review and editing.

**Jarno W. J. Huijs:** Writing - review and editing.

**Murillo Bellezzo:** Writing - review and editing.

**Rianne D. W. Vaes:** Writing - review and editing.

**Frank Aboubakar Nana:** Writing - review and editing.

**Koen J. Hartemink:** Writing - review and editing.

**Dirk K. M. de Ruysscher:** Writing - review and editing, Supervision.

**Lizza E. L. Hendriks:** Conceptualization, Writing - review and editing, Supervision.

## Disclosure

Dr. Hartemink reports outside of this manuscript personal fees as an invited speaker from AstraZeneca, MSD, and Bristol Myers Squibb. Institutional funding as a local principal investigator (PI) from KWF Dutch Cancer Society (ESLUNG study) and the Netherlands Cancer Institute - Antoni van Leeuwenhoek Hospital (UPLAN studies). Member guideline committees: Dutch guidelines on ‘SCLC’, ‘NSCLC’, ‘Mediastinal tumors’, and ‘Tobacco smoking cessation’. Board member of Dutch Thoracic Group and Chair scientific committee of Dutch Thoracic Group. Dr. Ruysscher reports outside of this manuscript research grant/support/Advisory Board: Institutional financial interests (no personal financial interests) from AstraZeneca, BMS, Beigene, Philips, Olink and Advisory Board: Institutional financial interests (no personal financial interests) for Eli-Lilly. Dr. Hendriks reports outside of this manuscript personal fees as an invited speaker from AstraZeneca, Bayer, Lilly, MSD, high5oncology, Takeda, Janssen, GSK, Sanofi, Pfizer (Inst), Medtalks, Benecke, VJOncology, Medimix (self); all payments were paid to the institution with the exception of Medtalks, Benecke, VJOncology, Medimix; fees paid to her institution for advisory board membership from Advisory boards: Amgen, Boehringer Ingelheim, Lilly, Novartis, Pfizer, Takeda, Merck, Janssen, MSD, Anheart, Bayer, AZ; institutional research grants from 10.13039/100004337Roche 10.13039/100004328Genentech, 10.13039/100004325AstraZeneca, Boehringer Ingelheim, Takeda, 10.13039/100004334Merck, 10.13039/100004319Pfizer, 10.13039/100004336Novartis, and 10.13039/100005564Gilead; institutional funding as a local principal investigator (PI) from AstraZeneca, GSK, Novartis, Merck, Roche, Takeda, Blueprint, Mirati, Abbvie, Gilead, MSD, Merck, Amgen; Member guideline committees: Dutch guidelines on NSCLC, brain metastases and leptomeningeal metastases (self), ESMO guidelines on metastatic NSCLC and SCLC (non-financial) Other (non-financial): secretary NVALT studies foundation, subchair of EORTC metastatic NSCLC systemic therapy, vice-chair scientific committee Dutch Thoracic Group. The remaining authors declare no conflict of interest.
